# Fermentation Gone Wild: A Biochemistry Laboratory Experiment

**DOI:** 10.1021/acs.jchemed.3c00499

**Published:** 2023-07-26

**Authors:** Julie T. Millard, Ronald F. Peck, Tina M. Beachy, Victoria L. Hepburn

**Affiliations:** ^†^Departments of Chemistry and ^‡^Biology, Colby College, Waterville, Maine 04901, United States

**Keywords:** Upper-Division Undergraduate, Biochemistry, Laboratory Instruction, Collaborative/Cooperative Learning, Hands-On Learning

## Abstract

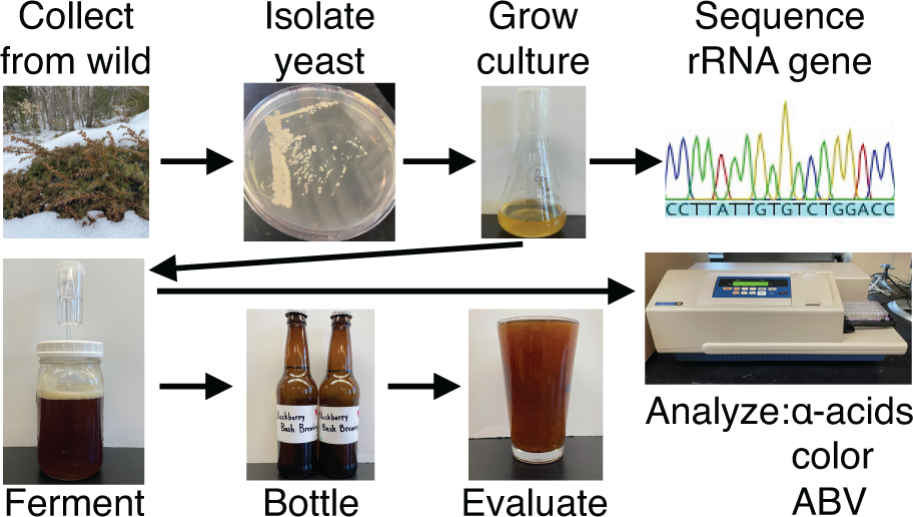

An experiment for the upper-level biochemistry laboratory is described in which students isolate a wild yeast from environmental sources and characterize the strain for its potential in the brewing industry. In addition to providing valuable experience in important biochemical techniques, this study also illustrates key principles of bioprospecting, the search for new biological sources with potential commercial or scientific value. By foraging for yeast in the wild, students explore the microbial diversity of their local environment and potentially find untapped sources of yeast that produce novel flavors and aromas. Overall, students engage with hands-on experience in bioprospecting, allowing them to appreciate the value of exploring biological diversity and its potential applications in the brewing industry.

## Introduction

Fermentation, a key metabolic pathway, is of great interest to students because of its role in making desirable food products such as bread, chocolate, sauerkraut, and alcoholic beverages. During fermentation, microbes convert sugars to lactic acid or ethanol, with secondary metabolites contributing to the overall flavor profile of the final product. Previous articles in this *Journal* have described both laboratory exercises^1−4^ and entire courses^5−7^ focused on fermentation. Several other papers have described beer brewing in undergraduate courses with *Saccharomyces* yeast from commercial or wild sources.^8−11^ One recent report described a semester-long laboratory project with a focus on microbiological techniques in which students isolated wild yeasts and used them to brew beer.^12^ We have expanded upon previous activities by developing and implementing a multiweek exercise in which biochemistry students use wild yeast collected *in situ* to brew beer. The students then perform several assays on the product and explore the metabolic ability of their yeast species to produce compounds that provide distinctive flavors. The identity of the yeast species is determined through DNA sequencing, and the beer is evaluated for its chemical properties and sensory profile through a variety of analytical techniques.

Commercial beer is most often fermented with one of two *Saccharomyces* species, generally *S. cerevisiae* or *S. pastorianus*. Both species have been domesticated since about the 16th century, optimizing their utility in the beer industry^13^ by maximizing production of ethanol and pleasant aromatic compounds while minimizing generation of toxins and “off-flavors”.^14^ However, some artisan breweries and vineyards use “open fermentations” that encourage the growth of wild microbes to create unique boutique-style beverages. For example, fermentation of Belgian lambic beers is initiated without any microbial starter inoculum, relying instead on microbes present in the brewery environment. Volatile compounds produced during spontaneous fermentation can impart interesting fruity and floral flavors and aromas to the product, with the presence of multiple yeast and bacterial species contributing to the ultimate sensory profile.

The goal of this project was to introduce students to the concept of “bioprospecting” in the context of the fermentation industry.^15−17^ Ethanol-producing yeasts can be isolated from a variety of sugar-rich environmental sources, with fruits, berries, and bark acting as natural reservoirs.^18^ While most people are familiar with brewer’s/baker’s yeast (*S. cerevisiae*), an estimated 150,000 species of yeast exist in nature, but only about 1% of them have been described.^19^ After isolating and identifying a wild yeast strain from its natural habitat, students characterize this strain for its potential utility in beer production. Aromatic components vary with the type of yeast introduced during the brewing process, leading to discernible differences in the resulting batches even when starting with the same initial components. During the multiweek process, students were able to harness the unique flavors and aromas of the wild yeast that they had collected, leading to some surprisingly pleasant brews. The exercise provided valuable lessons not only in biochemistry but also in the art and craft of beer science, which is increasingly interested in using non-*Saccharomyces* species to improve the diversity of the final product’s sensory profile.^20^

## Methods and Materials

This exercise was designed for a junior-level biochemistry course and requires three full laboratory periods to complete, with some brief preliminary work to culture the yeast prior to the first full week and minimal time to assess the final beer quality after it has been conditioned for a few weeks. Necessary equipment includes items standard to most biochemistry laboratories, including incubators, thermal cyclers, agarose gel units, power supplies, centrifuges, and spectrophotometers. Detailed procedures for students and notes for instructors are provided in the Supporting Information.

We initially developed this bioprospecting investigation during the height of the COVID-19 pandemic in conjunction with an “at-home” experiment to reduce the number of students in the laboratory. Students set up fermentation reactions with commercial yeast under controlled conditions to gain familiarity with achieving anaerobic conditions using food-grade airlocks and Mason jars. That experiment ran for 2 weeks, and during that time, students reported to the laboratory only to culture their wild yeast sample. Because of the high level of student engagement, we continued to refine and improve the bioprospecting exercise in subsequent years.

Initial preparation of the yeast sample requires a few brief trips to the lab over the course of a week, or these steps could be carried out during the previous 2 weeks in conjunction with another experiment. Students forage for a fruit, berry, or bark specimen that they add to liquid media containing sterile malt extract with antibiotics to select for yeast while limiting the growth of bacteria. Following incubation for about 2 days, the yeast culture is streaked for isolation onto an agar plate and grown until colonies appear in 2–3 days. Cultures and plates were placed in the refrigerator to minimize overgrowth until students returned to the lab. About 24 hours before the first full laboratory period, students inoculate a single colony in malt extract (without antibiotics) to produce the final culture used for DNA isolation and brewing.

The first full laboratory period takes about 3 hours to purify yeast DNA, to set up PCR reactions with fungal-specific primers for the 18S rRNA gene, and to begin an assay for phenolic off-flavors (POFs). Students also start the beer-making process in a food-safe space using a simple kit. The wort is prepared from commercial malt extract by adding sugar, water, and optional hops to a large cooking pot on a hot plate. After cooling, the mixture is placed in a 5-gallon carboy with spigot so that students can dispense the correct volume into a sterile Mason jar, add the yeast culture, and then cover with an airlocked fermentation lid. During the second laboratory period, which takes about 2 hours, students prepare PCR products for commercial sequencing to identify the yeast species and complete the POF assay. The third laboratory period is used for chemical analysis of the beer, which includes measuring IBU (bitterness),^21^ SRM (color),^6^ and ABV (alcohol by volume),^22^ and for bottling the beer. Four weeks later, the finished product can be assessed for aroma, flavor, and/or other sensory qualities. Our students used their results to prepare for a formal poster session in which they were expected to put their work into the context of the existing literature and potential commercial applications.

## Hazards

Students should wear gloves and protective eyewear throughout. Caution should be exercised with the hot plate, and the large pot containing hot wort should not be moved until it is sufficiently cool to handle. During the DNA isolation, students should avoid contact with the solution containing guanidine hydrochloride, which is hazardous in the case of skin contact, eye contact, or ingestion. Some of the reagents used for chemical analysis, including hydrochloric acid, isooctane, and tri-*n*-butyl phosphate, should be handled in a chemical fume hood and discarded in appropriate waste containers. Yeast plates and cultures were autoclaved prior to disposal.

## Results

Students first harvested wild yeast from specimens around campus, learning the importance of sterile techniques in the culturing and plating process. Even though we began this experiment during the Maine winter, students were able to successfully forage for a wide variety of different yeast sources from plants that they identified via smartphone apps such as iNaturalist. Students worked in pairs, allowing them to address their own experimental question through a strategic choice of yeast sources (e.g., same fruit, different location; different fruit, similar location).

**Figure 1:**
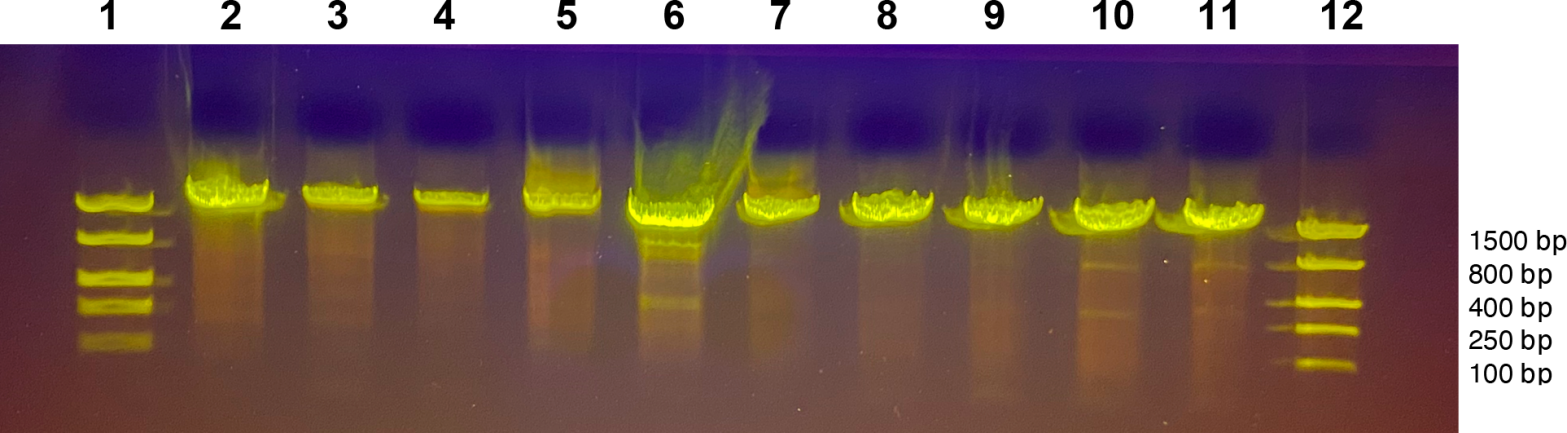
Amplified 18S rRNA PCR products from student yeast cultures obtained from Maine berries, fruits, and barks. Lane 1, size markers; Lane 2, blackberry (*Rubus allegheniensis*); Lane 3, rose hip (*Rosa canina*); Lane 4, crabapple (*Malus sylvestris*); Lane 5, juniper berry (*Juniperus communis* var. *depressa*); Lane 6, partridge berry (*Mitchella repens*); Lane 7, birch bark (*Betula papyrifera*); Lane 8, sour cherry (*Prunus cerasus*); Lane 9, winterberry (*Ilex verticillata*); Lane 10, buckthorn fruit (*Rhamnus cathartica*); Lane 11, oak bark (*Quercus montana*); Lane 12, size markers.

Successful DNA purification and amplification of the 18S rRNA gene yielded an approximately 1.6 kbp product^23^ (Figure 1). After PCR purification, products were commercially sequenced (Sanger method), which generally provides results within 24 h. Typical student success rate was about 80%, so instructors prepared backup samples through colony PCR, which allowed almost all students to obtain sequence data. Colony PCR is quick and cheap but relies on ethanol precipitation, a potentially challenging technique for students; therefore, they used commercial kits to prepare the yeast genomic DNA.

Fungal species were identified through a BLAST^24^ search of the DNA sequence on GenBank.^25^ A wide variety of yeasts were identified, with the same plant source often resulting in different yeast species. Somewhat surprisingly to our students, none of the isolates were brewer’s/baker’s yeast. However, despite the utility of *S. cerevisiae* in winemaking and brewing because of its high capacity for ethanolic fermentation, it does not commonly colonize the surface of fruits, even grapes.^26^ Nonetheless, our foraging efforts did yield several intriguing yeast strains with strong potential for craft brewing (Table 1), including *Metschnikowia pulcherrima*, an important species in winemaking because of its propensity to colonize grapes. This “killer yeast” reportedly has antimicrobial properties against other “spoilage” yeast species^27^ while also producing an array of fruity aroma compounds that enhance the overall flavor profile of wine and other beverages.^28^ We also successfully harvested *Wickerhamomyces anomalus*, which has been described as a promising candidate for production of low-alcohol beer and wine that retains good aromatic complexity.^29^ Another interesting find was *Lachancea fermentati*, previously reported in kombucha cultures.^30^ This unusual yeast produces both lactic acid and ethanol during fermentation, suggesting its utility to produce sour beers, which uniquely combine tart flavor with fruity and floral aromas. Many other species were virtually unknown in the brewing industry and varied in their potential to expand the array of useable yeast strains.

**Table 1:** Examples of Promising Yeast Strains Identified from a Variety of Local Foraged Local Sources

Yeast Species	Plant Sources
*Metschnikowia pulcherrima*	Crabapple, rose hip, juniper berry, buckthorn fruit, sour cherry
*Wickerhamomyces anomalus*	Juniper berry
*Lachancea fermentati*	Rose hip, oak bark
*Zygotorulaspora florentina*	Crabapple
*Aureobasidium pullulans*	Chokeberry, crabapple, rose hip, winterberry
*Hanseniaspora uvarum*	Blackberry, crabapple
*Zasmidium cellare*	Juniper berry, partridge berry

Each student set up a fermentation reaction with a commercial beer mix in a Mason jar, inoculating the wort with a small portion of the overnight yeast culture. Cultures that smelled musty or otherwise unpleasant were not used for fermentation; therefore, instructors had extra cultures available for students to use. Fermentation was also initiated with commercially obtained brewer’s yeast for quality-comparison purposes. Jars were sealed with an airlock and allowed to ferment in the dark over 2 weeks. In the subsequent laboratory session, a variety of objective measures of beer character were determined. Beer bitterness, percent alcohol, and sensory notes varied by yeast, even when using the same wort (sample data from one lab section are shown in Table 2). The palatability of the final conditioned beer also varied considerably, clearly demonstrating the crucial role that the yeast plays in the final properties of the beer that they produce.

**Table 2:** Fermentation of the Same Beer Wort by Different Wild Yeast Strains Isolated from Central Maine

Source	Yeast Species	Culture Aroma	IBU	ABV	POF+	Sensory Notes	Rating^a^
Rose hip	*Aureobasidium pullulans*	Smoky, honey	6.9	1.6	Yes	Floral/spices	3.6
Crabapple	*Zygotorulaspora florentina*	Apple cider	6.5	0.79	No	Fruity	3.9
Winterberry	*Aureobasidium pullulans*	Smoky, honey	3.3	4.1	Yes	Floral/spices/chocolate	4.2
Oak bark	*Lachancea fermentati*	Fruity, floral	4.4	2.9	No	Floral/fruity, sour	4.0
Birch bark	*Papiliotrema laurentii*	Woodsy, earthy	5.3	4.4	Yes	Spices/caramel	2.6
Buckthorn fruit	*Metschnikowia pulcherrima*	Fruity, crispy, earthy	5.9	4.5	ND^b^	Fruity/caramel	4.0

a Volunteers of legal drinking age assessed the final product on a scale of 1 (poor) to 5 (excellent). With *S. cerevisiae* as a reference, the final product earned a rating of 3.0, with comments such as “bland”.

b Not determined.

In addition to identification through sequencing, students characterized their yeast strains for the potential to produce phenolic compounds, which are considered by some to be “off-flavors” but are favored in some types of beer. Most lager and ale yeast strains are negative for phenolic off-flavors (POF−), while witbier and hefeweizen strains are positive for phenolic off-flavors (POF+), which generate a characteristic clove-like or smoky aroma and flavor.^31^ Several of the brews with *Aureobasidium pullulans*, a POF+ strain, were described as having an aroma of “spices” or “smoke”, supporting the biochemical tests.

## Discussion

In this laboratory exercise, students successfully isolated, characterized, and brewed beer with different yeast species from environmental samples, reinforcing several fundamental skills in biochemistry along the way. Overall, there was a high level of engagement with this project, and students particularly appreciated the tangible and relatable applications and timeliness with regard to bioprospecting. Increasing the representation of new strains from the immense reservoir of wild yeast isolates is a current trend in the craft beer industry.^32^ Brewers are increasingly using mixed starter cultures of *S. cerevisiae* and non-*Saccharomyces* species to enhance the flavor, aroma, and bouquet of fermented beverages.^20^ While our students set up fermentations with only one yeast species, an intriguing follow-up study would be to inoculate first with a local wild strain and later introduce brewer’s yeast to potentially raise the ethanol content.

Anticipated learning outcomes include gaining expertise with fundamental laboratory protocols (e.g., sterile techniques, DNA purification, PCR, and bioinformatics) and an increased understanding of the process of fermentation and the wide diversity of yeast metabolism. Students in the most recent iteration of the experiment rated it 4.6 out of 5 possible points in terms of enjoyment and made significant gains in their understanding of core principles, as demonstrated by prelab and postlab assessment (Table 3; also see the notes for instructors). One notable comment that reflected the general consensus was, “Super cool to see what can be done with yeasts from just outside our doors.” Furthermore, the high quality of the final posters demonstrated that student engagement with the experiment’s theme led to improved motivation and learning. Students effectively used primary literature to contextualize their work, recognized that all yeasts are not created equal, and suggested appropriate follow-up studies based on their data. While initiatives for locally sourced ingredients in home and craft brewing have focused mainly on grain and hops,^17^ this experiment highlights the potential of cataloging local yeast strains to develop a unique signature flavor profile.^33^

**Table 3:** Pre- and Postlab Gains in Understanding Assessed in 2023 (*n* = 15)

Question	Pre-Lab (% Correct)	Post-Lab (% Correct)
Which of the following conditions will likely result in the most alcohol production assuming that the same amount of sugar and yeast are present in each case?	47	87
What was likely the source of yeast used in the earliest fermentations carried out by prehistoric humans?	53	100
What molecule(s) contain the carbons from sugar after yeast fermentation?	27	80
Different types of beer have very different flavors and aromas. List the ingredients of fermentation that may affect the flavor profile.	47	93
What are the chances that viable yeast can be found outside during a Maine winter?	67	100

## Supporting Information Available

Notes for Instructors (PDF, DOCX)
Student Handout (PDF, DOCX)
Sample Poster #1: Backyard Brewery: Brewing Beer with Wild Yeast (PDF)
Sample Poster #2: Brewing the Perfect Beer: Exploring the Effects of Yeast Strains on Flavor, Aroma, and Alcohol Content (PDF)
