# Supplementary material for: Fermentation Gone Wild: A Biochemistry Laboratory Experiment
Source: J Chem Educ. 2023 Jul 26;100(8):3076–80. doi: 10.1021/acs.jchemed.3c00499 (PMC10413941; doi:10.1021/acs.jchemed.3c00499)
Supplement: Supplementary file 2 — ed3c00499_si_002.docx [file ed3c00499_si_002.docx]

Supporting Information

Fermentation Gone Wild: A Biochemistry Laboratory Experiment

Julie T. Millard*^1^, Ronald F. Peck^2^, Tina M. Beachy^2^, and Victoria L. Hepburn^1^

Departments of Chemistry^1^ and Biology^2^, Colby College, Waterville ME 04901

*e-mail: [jtmillar@colby.edu](mailto:jtmillar@colby.edu)

Notes for Instructors

**Materials Needed**

- Dried Malt Extract (Muntons Spraymalt Light or similar) ~$10 for 1 pound at brewing supply store or online. 1 pound is enough for ~400 students
- Liquid Malt Extract; There are many available for brewing batches of ~5 gallons for ~$25.
- Hops (1 package for ~$5) It’s also possible to buy pre-hopped liquid malt extract. We usually add a few hops to the wort while it’s boiling.
- Beer Bottles (~$24 for case of 24)
- Bottle caps (~$10 for pack of 100)
- Beer Bottle capper (~$20)
- Mason jars – 1 quart (~$30 for pack of 8)
- Lids and airlocks for jars (Year of Plenty BPA-Free, Clear Fermentation Lids for Making Sauerkraut in Wide-Mouth Mason Jars; ~$18 for pack of 4)
- Cooking pot large enough to prepare the wort (i.e., IMUSA Stainless Steel Stock Pot 20 Quart, ~$35)
- Hot plate large enough to boil water in the pot
- Cooler with spigot to distribute wort into jars (i.e., Igloo 10 Gallon Seat Top Water Jug With Cup Dispenser, ~$60)
- Ethanol, 96-100%
- Ampicillin (CAS 69-53-4), 50 mg/mL stock in 70% ethanol, dilute 1000X in media
- Chloramphenicol (CAS 56-75-7), 10 mg/mL in 70% ethanol, dilute 1000X in media
- Sorbitol (CAS 50-70-4)
- EDTA (CAS 60-00-4)
- Beta-mercaptoethanol (60-24-2)
- Lyticase enzyme (Millipore Sigma; Cat. No. 04963; 1 g @ $52)
- Ferulic acid (CAS 537-98-4)
- Hydrochloric acid, 3 M (CAS 7647-01-0)
- Isooctane (2,2,4-Trimethylpentane, CAS 540-84-1)
- Tributyl phosphate (CAS 126-73-8)
- Sulfuric acid (CAS 7664-93-9)
- Potassium dichromate (CAS 7778-50-9), make 10% solution in 5 M sulfuric acid
- DNeasy Blood & Tissue Kit (Qiagen; Cat. No 69504; 50 preps for $215)
- QIAquick PCR Purification Kit (Qiagen; Cat. No. 28104, 50 preps @ $141)
- Phusion Hot Start Flex 2X Master Mix (New England Biolabs; M0536S [100 reactions @ $227]). Other polymerases likely work, but we have the most consistent results with this one.
- Fungal 18S universal primers (nu-SSU-0068-5´-20 – CCATGCATGTCTAAGTWTAA; nu-SSU-1647-3´ - ANCCATTCAATCGGTANT) (IDT DNA; purchase the smallest scale possible). Upon arrival, DNA pellet should be dissolved in 10 mM Tris, pH 8.0 to achieve a 100 µM stock solution for long-term storage (add 10x the number of nanomoles in µL of water). Working dilutions of 10 µM should be made in sterile dH2O and stored at –20 °C, or in the refrigerator for up to 2 weeks.
- Lonza Flash Gels, 1.2% (Cat. No. 57023 [1 box of 9 gels @ $156]) (Note: traditional agarose gel electrophoresis can also be performed but takes longer to run.)
- FlashGel Loading dye (Lonza; Cat. No. 50462; @$188)
- DNA ladder to allow quantification of ~1.6 kb expected PCR products for example, FlashGel Quantladder (Lonza; Cat. No. 50475 @ $195 for 50 uses)
- Lonza Flash gel systems and power supplies (or conventional agarose gel systems)
- Sterile deonized water
- Thermocycler
- UV/Vis spectrometer and appropriate cuvettes
- 1.5-mL microcentrifuge tubes, PCR tubes, micropipetters and tips, serological pipets and pipet-aid or bulb
- Heat block, vortex mixers, microcentrifuge, centrifuge for 96-well plates (or samples can be transferred to microcentrifuge tubes to pellet yeast for the ferulic acid assay)
- Clear, flat-bottom sterile 96-well plates
- Plate Reader

**General Notes**

- Students were expected to read the laboratory handout and write appropriate procedures into their laboratory notebook before class. No pre-lab lecture was given.
- To prevent the growth of contaminating bacteria, antibiotics (ampicillin and chloramphenicol) were included in the liquid media used for the initial growth from the plant source and the plates used for yeast isolation. These antibiotics were not included in the yeast culture that was used for fermentation to avoid any issues with allergies to these antibiotics.
- For genomic DNA extractions, we used the DNEasy Blood & Tissue Kit and followed the supplemental protocol for yeast from the Qiagen website. This protocol requires sorbitol buffer (1 M sorbitol, 100 mM EDTA, 14 mM beta-mercaptoethanol, can be stored at 4 °C for at least one week) and the lyticase enzyme that are not included with the kit.
- We had the students sequence their PCR products in both directions to increase the chances of getting usable sequence for identification. These sequences were then aligned in Geneious, but any alignment tool (many available online) may be used.
- As a backup to the students’ PCRs, instructors set up a colony PCR to amplify the fungal 18S rRNA gene for sequencing:
  - Single colonies were picked into 100 µL of 0.2 M lithium acetate/ 1% SDS and incubated at 70 °C for 15 min to lyse the cells.
  - Cell debris was pelleted in a microcentrifuge at max speed for 3 min
  - Supernatant was removed to a fresh tube
  - 300 µL of 100% ethanol was added to precipitate the DNA
  - DNA was pelleted in a microcentrifuge at max speed for 5 min
  - Supernatant was discarded, and the DNA resuspended in 30 µL TE.
  - 5 µL was used as template in a PCR described in the lab handout.

**Pre- and Post-Lab Assessment**

Pre-Assessment contained this header on a Google Form:

*You must complete this survey before reading the lab handout for Experiment 3. It is due by 5:00 on Friday, Feb 17. Do not look up the answers to these questions. We are gauging how much you already know about fermentation before doing this experiment.*

Post-Assessment contained these questions on the final exam.

**1.**  Which of the following conditions will likely result in the most alcohol production assuming that the same amount of sugar and yeast are present in each case?

a) A sealed container that allows no gas in or out

b) A container open to the air

c) A container open to the air and stirred to increase aeration

d) A container sealed except that gas can escape but not be brought in

e) A container sealed except that gas can be brought in but not escape

**2.** What was the likely source of yeast used in the earliest fermentations carried out by pre-historic humans?

**3.** What molecule(s) contain the carbons from sugar after yeast fermentation?

**4.** Different types of beer have very different flavors and aromas. List the ingredients of fermentation that may affect the flavor profile.

**5.** What are the chances that viable yeast can be found outside during a Maine winter?

a) Slim to none

b) It depends on how bad the winter is

c) Yeasts are ubiquitous, no matter the season

Answers:

1. d

2. wild yeast growing on fruiting bodies, bark, and other plant materials

3. ethanol, CO_2_

4. yeast species (also type of sugar, hops, presence of oxygen, pH)

5. c

**Poster Session Guidelines**

- General overview— Working with a partner, you will present a poster based on Experiment 3 (*Wild Times with Wild Yeast*) during the last week of lab. Create your poster as a single 48 x 42 Powerpoint slide, with information flowing from top to bottom and from left to right. There are many examples of posters throughout Keyes and Arey, so use these examples to get some ideas of how to achieve an effective poster layout (and perhaps how not to!).
- Poster Content—Your poster should include the following:

1) The TITLE, which should be as descriptive, unique, and catchy as possible

2) The names of the AUTHORS and their AFFILIATION(S).

3) The INTRODUCTION, which defines the goals of the work and its literature context. Note that you have a large amount of leeway on how to present your work, but you should clearly state an interesting question that you were trying to answer.

4) The METHODS section, which should be a pictorial flow chart.

5) The RESULTS section, which presents key data and analysis, including relevant figures and tables.

6) The CONCLUSIONS section, which summarizes your key findings, how they fit into context of the literature, and future directions for the work.

7) The REFERENCES section, which contains all the sources used to set the context of your work. You can use any format for references that you wish, but be consistent.

8) The ACKNOWLEDGEMENTS section, which is where you thank those who contributed to the work.

- General format—Tips for formatting are as follows:
  - Try to avoid a 'wall-of-text'. Remember that posters are a visual format for presenting data; therefore, focus on figures and use bullet points as much as possible to highlight key introductory material, results, and conclusions. Your goal is to make it as simple as possible for your audience to quickly grasp the key points of your poster.
- Appropriate font sizes are in the range of 24-36 point for text, 48-60 for headings, and 76-90 for titles. Experiment with font size before printing the final poster.
- Images will be blown up a lot, so they should be saved in a high-resolution format. High-resolution TIFFs and JPEGs work fairly well, but if you take a small picture from the web, it is likely that it will become pixelated when blown up and look terrible.
- Avoid fancy backgrounds and fonts, which often cause failure during printing, as well as using up a lot of ink.
- Make sure that you have printed your poster on a color printer and checked it over carefully before initiating poster printing, which is very expensive.
- Presenting your poster— You and your partner will prepare a brief oral synopsis of your work to present to the group as it circulates to your poster. Keep it less than 5 minutes— highlight your goals, key findings, and why they are important. Design your overview as a “sound bite” that captures your main points in a succinct and compelling fashion. Keep your methods to an absolute minimum, as everyone in the audience should be well versed in what you did in lab, since they did the same things themselves!
- Evaluation— Your posters will be evaluated by your instructors using additional evaluative comments from the other students in the course as well as any special guests and other attendees. The following criteria will be used to evaluate your posters:

1) **Visual impact** — Is the poster laid out well? Are all graphics and text of

appropriate size and quality? Does the poster grab the viewer’s interest with interesting graphics and pictures? (A picture is worth a thousand words!) Is the color scheme attractive?

2) **Content** — Is your key research question clearly stated? Is the treatment of the work at an appropriate level for the audience (biochemistry students, faculty, and other interested parties)? Does the information in the poster flow logically? Are the data and analysis complete, yet concise? Are relevant and timely references included? Are the conclusions and future directions insightful? Did you appropriately credit the contributions of collaborators?

3) **Presentation** – Did you explain your poster effectively during the poster session? Were you able to answer questions effectively? Did you stick to your allotted time (5 minutes)?

**Wild Yeast Poster Rubric**

**Visual Impact**

2 Effective layout grabs the viewer’s interest. All graphics and wording are of appropriate size and quality.

2 Bullet points with minimal text.

2 Suitable and effective visuals, including pictures of yeast source and highlights of the process.

2 Logical flow to the content on the page.

2 Attractive color scheme.

**Content**

5 Title is informative, catchy, and relevant to the work (not just the title of the lab)

15 Introduction explains the motivation and importance of the work at a level appropriate for the audience. The experimental question is explicit, sophisticated, and makes sense. The work is set in the context of the literature.

10 Methods is an easy-to-follow flowchart with pictures.

20 Results— data and analysis, with relevant figures and tables, includes the following:

- Source of yeast
- Yeast ID with % identity
- Ferulic acid test
- IBU
- ABV
- SRM
- Subjective ratings of final product

10 Conclusions— thoughtful and relevant summary of the findings, how they fit into context of the literature, and future directions.

10 References— ~5 relevant, somewhat recent, primary literature references were appropriately used and cited.

5 Acknowledgements— appropriate credit was given to those who made the work possible, including classmates who provided data.

**Presentation**

10 “Elevator pitch”— Key findings were adequately summarized during the poster session in the allotted time (5 minutes per poster), and questions were answered effectively.

5 Overall professionalism and quality of product; no more than one spelling/typographical error

**_____Total points (out of 100)**
