# Supplementary material for: Fermentation Gone Wild: A Biochemistry Laboratory Experiment
Source: J Chem Educ. 2023 Jul 26;100(8):3076–80. doi: 10.1021/acs.jchemed.3c00499 (PMC10413941; doi:10.1021/acs.jchemed.3c00499)
Supplement: Supplementary file 3 — ed3c00499_si_003.pdf [file ed3c00499_si_003.pdf]

Supporting Information

Fermentation Gone Wild: A Biochemistry Laboratory Experiment

Julie T. Millard*[1], Ronald F. Peck[2], Tina M. Beachy[2], and Victoria L. Hepburn[1]

Departments of Chemistry[1] and Biology[2], Colby College, Waterville ME 04901

*e-mail: jtmillar@colby.edu

Student Handout: Fermentation Gone Wild

Experimental Background

During the early stages of the pandemic, certain products were unavailable at the supermarket. Perhaps surprisingly, Baker's yeast was one of those items due to a surge in "comfort baking." One result of this shortage was a rise in the popularity of sourdough starter, a fermented mixture of flour and water inhabited by a colony of "wild" microorganisms called a SCOBY (Symbiotic Culture Of Bacteria and Yeast). Wild yeast in the starter cause the dough to rise through production of carbon dioxide via ethanolic fermentation, while lactobacilli produce the lactic acid that gives the bread its characteristic tartness. No two SCOBY's are identical, with geographical variation depending on the local microbes, feeding schedule of the starter, and incubation conditions.

Beer making also takes advantage of yeast metabolism, starting with a mixture of malted barley (the sugar) and hops (for flavor and aroma) in water. Commercial beer is most often fermented "single culture" style, with one of two kinds of yeast: *Saccharomyces cerevisiae,* favored in ales, or *Saccharomyces pastorianus*, favored in lagers. However, as with sourdough bread, it is possible to brew alcoholic beverages with wild microbes, a handy skill to have during the next lockdown! In fact, some breweries and vineyards run "open fermentations" that encourage the growth of wild microbes to create beverages with novel flavors. Volatile aroma compounds produced during spontaneous fermentation can impart interesting fruity and floral flavors to the product, with the presence of multiple yeast, other fungi, and bacterial species contributing to a unique final flavor profile.

In this experiment, we are going to try some "backyard yeast wrangling." Our goals include:

- Harvest local wild microorganisms to create a microbial culture. (We will add antibiotics in order to minimize the chances of any bacteria crashing the party!)
- Isolate and grow up one of the yeast strains in the culture.
- Identify this yeast strain through DNA sequencing.
- Assess the isolated wild strain for its potential to yield a palatable beverage in comparison to commercial yeast strains.
- Identify variables in the brewing process that influence the final flavor profile.
- Put this work into the context of the literature during a final poster presentation.

Experimental Procedure

Week 1.

Part A. Wild Yeast Wrangling

The microbes that live on fruits are primarily yeasts and other fungi. Interesting sources of wild yeast around campus could include chokecherry, rose hip, crabapple, juniper berry, or a small piece of bark (e.g., from an oak tree). Each student will perform this part of the experiment individually, but you should consult with your partner so that you are testing complementary sources.

1. Do some reconnaissance before lab to locate an interesting potential yeast source (fruit/berry/bark). Identify the plant— there's an app for that— and record your choice on the shared Google form linked on the lab page.
2. Pick up a baggie before heading out. Put it on your hand inside out, collect a single small fruit/piece of bark, and flip the bag right-side out before sealing it. You could also swab a larger fruit and place the swab inside the tube.
3. Record GPS coordinates of the location and take a picture of the source plant.
4. Bring the sample to the lab between 1:00 and 1:30 on your lab day.
5. Transfer your sample to a culture tube containing growth medium. Make sure that you do not overly stuff the tube with your sample—microbes are small. Furthermore, if your tube has too much plant material in it, it is likely to absorb all the water in the medium and dry everything out, thereby preventing microbes from growing.
6. Your instructor will place the culture tube in the 30°C incubator for about 2 days until it is cloudy. Microbes should be happily growing by the next time you come to lab.

Week 2.

Part B. Plating Wild Microbes

Your culture tube should be chock full of microbes. Mixed wild cultures have long been used by brewers and are still popular among some home winemakers. This method can certainly lead to some tasty beverages. However, for better reproducibility in the brewing process, it's necessary to isolate individual pure strains. We will set up fermentations with a single isolated microbial strain for comparison to commercial yeast and to see if we have any interesting local wild strains for the LYP Culture Bank.[1] Review the streaking protocol here before coming to lab: https://microbenotes.com/streak-plate-method-principle-methods-significance-limitations/ (see figure below).

1. Put on a fresh pair of gloves and maintain good sterile technique throughout. Obtain an agar plate containing yeast nutrients and antibiotics to discourage bacterial growth.
2. Pass a streaking loop through a Bunsen burner then touch the loop to the agar to cool it.
3. Place the loop inside your culture tube and then streak it lightly across the agar plate. Sterilize the loop again, and then use the first streak to streak again…and the second streak to streak again. You are trying to spread out the microorganisms on the plate.
4. Incubate the agar plate at room temperature in your lab drawer. Check on your plate over the next few days and place it in the refrigerator when you can see discrete yeast colonies. This will take about 2 days. Take a picture of your plate.
5. ***Return to lab 24 hours before your lab time next week***, and making a note of which colony you took, start an overnight culture in a sterile flask containing 60 mL growth medium as follows: Flame the mouth of the flask, pick up a colony on your plate with a yellow pipette tip, transfer it to the flask, flame the mouth again, cover with foil, label the flask with a Sharpie. Your instructor will place it in the incubator.

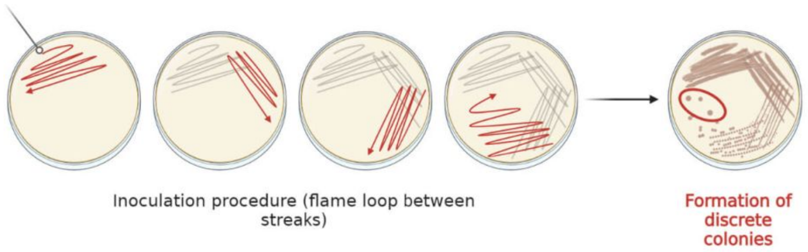

[1] https://bootlegbiology.com/about-2/become-a-contributor/

Week 3

Assess the quality of your culture through a smell test before proceeding. Record all noticeable aromas and how strong they are. While aroma is a subjective property, it is unlikely that a yeast that smells like feet is a good candidate for beer making.

- Promising aroma descriptors include fruity, floral, cloves/spices, smoky, earthy, yeasty, astringent (like rubbing alcohol), and sweet (like honey or maple syrup). Be as specific as possible…for example, "like apples" rather than just "fruity."
- Aroma descriptors that might indicate a yeast should be eliminated from further testing include musty or like feet.

Each pair of students should perform two fermentations. If both wild cultures seem promising, then proceed with both cultures for the rest of the experiment. If one (or both) seem like duds, then other options are commercial yeast or cultures prepared by your instructors.

Part C. DNA Isolation

We are using the DNeasy Blood & Tissue Kit from Qiagen.

1. Place 1.5 mL of your yeast culture into a microfuge tube.
2. Centrifuge for 5 min at 8000 rpm (5000 $\times g$). Remove and discard the supernatant.
4. Add 600 µL sorbitol buffer to the tube and pipet up and down to resuspend the cells.
5. Add 200 µL (200 units) lyticase enzyme and incubate at 30°C for 30 min. (The lyticase enzyme cleaves glycosidic bonds in the carbohydrates that make up the yeast cell walls.) Proceed to part E while you are waiting.
6. Centrifuge the lysed cells, now called "spheroblasts," for 5 min at 4500 rpm (1000 $\times g$). If your pellet is too snotty, re-spin at a slightly higher speed.
7. Discard supernatant and resuspend the spheroblasts in 180 µL Buffer ATL.
8. Add 20 µL proteinase K and vortex.* (*All vortex steps should be done for 10-15 sec.)
9. Incubate at 56°C for 15 min, vortexing about half way through and again at the end of the incubation.
10. Add 200 µL Buffer AL to the sample and mix thoroughly by vortexing.
11. Add 200 µL ethanol and mix thoroughly by vortexing.
12. Pipette the mixture (including any precipitate) into the DNeasy mini spin column.
13. Centrifuge at maximum speed for 1 min. Discard the flow-through and collection tube.
14. Place the spin column into a new collection tube and add 500 µL Buffer AW1.
15. Centrifuge at max speed for 1 min. Discard the flow-through and collection tube.
16. Place the spin column into a new collection tube and add 500 µL Buffer AW2.
17. Centrifuge at max speed for 3 min. Discard the flow-through and collection tube.
18. Place the spin column into a new <u>microfuge tube</u> for elution and add 100 µL Buffer AE.
19. Incubate at room temperature for 1 min.
20. Centrifuge at max speed for 1 min. <u>SAVE the flow-through as it contains your DNA.</u>

Part D. Amplification of the 18S rRNA Gene

To identify our newfound fungal friends, we will sequence the 18S ribosomal RNA gene. We will use "degenerate" primers that have a mixture of bases at certain positions. This property allows them to have a wide range of binding sites so that they can be used to amplify the 18S rRNA gene from any (known) fungus.

<u>*PCR Recipe per reaction: multiply by 2.5x to create enough for you and your lab partner!*</u>

25.0 µL Phusion polymerase master mix (contains buffer, Mg^{2+}, dNTPs, DMSO, Phusion polymerase)
17.0 µL sterile dH_2O
3.0 µL forward/ reverse primer mix (at 10 µM)

1. Add 45.0 μL of Master Mix and 5.0 μL of each template DNA to separate PCR tubes. Deliver these solutions into the bottom of each tube, and mix with the pipet tip after adding template DNA.
2. Tap the tubes down on the bench to remove bubbles.
3. Make sure that the tubes are firmly closed and labeled. Put your initials on the side and top.
4. Place the tubes in the thermal cycler following the "Fungi18S" program: 94 °C / 4 min; 30 cycles of (94 °C / 30 sec, 56 °C / 60 sec, 72 °C / 90 sec); 72 °C / 10 min; 8 °C hold.

<u>Primer information</u>

Forward primer: nu-SSU-0068-5´-20 - CCATGCATGTCTAAGTWTAA

Reverse primer: nu-SSU-1647-3´ - ANCCATTCAATCGGTANT

Part E. Potential for Phenolic Off Flavors

The flavor profile of a beer arises through the production of volatile secondary metabolites that vary depending on the yeast strain. Some metabolites contribute pleasant fruity or floral aromas, whereas others smell unpleasant. Styles such as Belgian witbier and German hefeweizen are deliberately brewed with yeast strains that generate phenolic compounds that are considered "off flavors" in other beer types. An example of a yeast-derived "off flavor" is 4-vinyl guaiacol (4-VG; **A**), which has a distinctive smoky, clove-like aroma and is derived from ferulic acid (**B**) during fermentation.

In this part of the project, you will assess the capacity of your yeast strain to convert ferulic acid to 4-VG. A yeast strain with this capacity would be bettered suited for production of a witbier or hefeweizen. We will use one 96-well plate for each pair of students.

1. Add 25 μL of your yeast culture to 175 μL growth medium spiked with ferulic acid (100 mg/L) in a 96-well plate.
2. Repeat in two other wells—you now have three identical samples of your yeast on the plate.
3. Set up three blanks containing 25 μL regular growth medium (no ferulic acid or yeast) added to 175 μL spiked growth medium.
4. Set up three wells inoculated with 25 μL of a cultured POF+ strain and 175 μL ferulic-acid- spiked growth medium as the positive control.

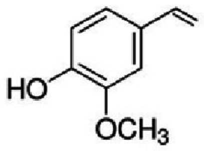

(A)

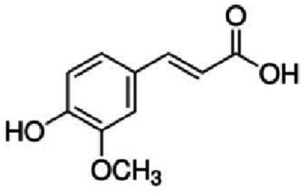

(B)

5. Similarly, set up three wells inoculated with a POF- strain as the negative control.
6. Seal the plate tightly, then incubate on the rocking platform until next lab period.
7. When you return to lab during Week 4, centrifuge the plate for 3 min (or transfer each sample to a microcentrifuge, then centrifuge.)
8. Transfer 100 μL of each supernatant to a new 96-well plate. Be careful to avoid the yeast pellet when removing the supernatant.
9. Add 100 μL of deionized water to each well to dilute the samples.
10. Measure absorbance at 325 nm using the plate reader.
11. Strains are considered POF+ if the measured amount of ferulic acid is below the 90% confidence interval of the blank. Prepare a table with these data and your analysis in your notebook for next week.

Part F. Fermentation

1. Sterilize the cooking pot, Igloo cooler, and your Mason jars with a dilute solution of Star San. (We don't want any bacteria doing biochemistry that might negatively impact the taste of our craft beer!) Let this solution sit inside the vessel for about 5 minutes, then rinse three times with sterile water.
2. Open the top of the can of malt. Place the can into a warm-water bath (~50 °C) for ~10 minutes to reduce the malt's viscosity.

3. Pour 1 gallon (3.7 L) of water into the cooking pot and heat on the hot plate in the food-safe room until it is warm to the touch. While it is heating, add hops extract, table sugar, or other ingredients as specified by the recipe of the mix for your lab day. Stir with a clean wooden spoon until the mixture dissolves.
4. Remove the cooking pot from the hot plate. Pour in the malt. Rinse the malt can repeatedly with some of the second gallon of water until the can is fairly clean, pouring all this water into the pot. Add the remaining water (3 gallons total) to the pot while stirring until the mixture is homogeneous.
5. Pour the mixture (the “wort”) into the Igloo cooler.
6. Swirl your remaining culture to suspend the yeast, then pour ~50 mL into a 1-quart wide-mouth Ball jar. Label jars with yeast type, your initials, and date. Each student should use one jar.
7. Use the spigot to dispense ~700 mL of wort into your jar.
8. Seal the jar with the lid and airlock. Be sure to put some water in the airlock to prevent air from entering the wort. (Why is it important to limit oxygen during this process?)
9. Let the jars ferment for a couple of weeks in the dark at room temperature.

Week 4. *Note: Remember to finish up Part E today, but get started with Part G upon arrival.*

Part G. Assessment and Purification of PCR Products

1. Check the quality and quantity of your PCR products on a 1.2% FlashGel. Place 1 µL of each PCR reaction into a fresh microcentrifuge tube and add 3 µL of dH_2O and 1 µL of loading dye. Centrifuge the samples briefly to mix.
2. Load each sample into its own well on the gel, saving one of the middle lanes for the marker, which is 5 µL of the FlashGel QuantLadder (see image below). Run the gel at 250 V for about 7 minutes, or until the ladder has resolved.
3. After your gel has finished electrophoresis, take a picture of it.
4. For successful sequencing, the product should be at least as bright as the 400 bp marker.
5. If your amplification was successful, proceed as follows. Otherwise, your instructor may have another PCR reaction from a colony PCR prepared from your plate for you to use.
6. Transfer the PCR reaction to a microcentrifuge tube and add 250 µL of PB Buffer (5x the sample volume). (This buffer contains guanidine hydrochloride; avoid contact!)
7. Pipette up and down to mix your sample, and then transfer the entire mixture to a Qiagen clean-up column (QIAquick column).

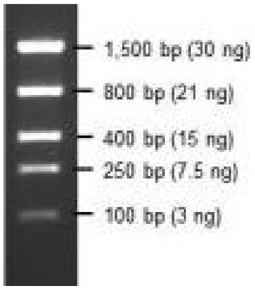

8. Centrifuge at maximum speed for 1 minute.
9. Discard the flow-through into the specified waste container and replace the column in the collection tube.
10. Add 750 µL of PE buffer to the clean-up column.
11. Centrifuge at maximum speed for 1 minute.
12. Discard the flow-through into a waste container and replace the column in the collection tube.
13. Centrifuge at maximum speed for 1 minute, again, in order to remove residual ethanol in the column left over from the PE buffer.
14. Discard the flow-through into the specified waste container and place the column into a fresh microcentrifuge tube.
15. Add 30 µL of sterile dH_2O to the center of the white disc inside the column. Let it sit for 1 minute at room temperature.
16. Centrifuge the sample at maximum speed for 1 minute to elute your DNA. Discard the column.

Part H. Preparation for Sequencing

We are sending out our PCR products to Eurofins MWG Operon for Sanger sequencing, which requires purified PCR product at a concentration of at least 20 ng/μL.

1. Prepare two samples as follows: To 8 μL of purified PCR product in a microcentrifuge tube, add 4 μL of primer (at 2 μM) and centrifuge the samples briefly to mix. One sample contains the forward primer; the other sample contains the reverse primer.
2. Put a barcode on each tube, recording the barcode assigned to each sample on the whiteboard.
3. Your instructor will share the class sequencing results when they are available.
4. Perform the analysis in Geneious Prime as follows.
7. File-Import-Files: choose Chromatogram format, then import the abi sequencing files.
8. Do this for both the forward and reverse sequence.
9. Select both sequences by checking the box to the left and choose Tools…Align/Assemble…Pairwise Align and check box that says "Automatically determine direction."
10. Select the region that contains the best consensus between sequences (as green as possible in the overlapping region).
11. Go to https://blast.ncbi.nlm.nih.gov/Blast.cgi and click on "Nucleotide BLAST."
12. Copy the consensus sequence from Geneious Prime and paste it into the box.
13. For "Database", choose the option "rRNA/ITS databases."
14. In the pulldown menu for Database, choose "18S ribosomal RNA sequences (SSU) from Fungi type and reference material."
15. Leave all of the other settings as the default and click the "BLAST" button.
    - You will get a list of sequences that your sequence is most similar to. Choose the top search hit and note its "Query cover" (percent of your sequence length that aligns with the sequence in the database) and percent identity (percent of your sequence within the alignment that is identical to the sequence in the database).
16. Compile your results in your notebook for next week.

Week 5

Part I. Assessment #1- Objective Measures of Beer Character

There are several quantitative parameters of beer character, including alcohol content (ABV), bitterness, and color, all of which we can measure in lab. There are also several subjective parameters that depend on personal preference and tastebud genomics! We will measure some objective parameters today and save the subjective evaluation for later, when our beer has appropriately aged.

1. Beer Bitterness (IBU)

One important parameter of any beer is bitterness. Although taste is subjective, and flavor is a balance of many ingredients, the brewing industry has developed an objective "bitter" scale using International Bitterness Units (IBU). Most beers have a measured IBU between 5 (low bitterness) up to 120 (high bitterness).

The IBU value is actually a measurement of certain chemical compounds that lead to the characteristic bitter flavor of many beers, including organic acids and polyphenols. Hops (*Humulus lupulus*) contains water-insoluble alpha acids that isomerize during boiling to produce iso-alpha-acids, which are strong contributors to a beer's bitterness. IBU can be determined by organic extraction of the iso-alpha-acids and spectrophotometric measurement of the resulting extract. The relationship between IBU and iso-alpha-acid is that 1 IBU ≈ 1 mg/L of iso-alpha-acid. Obtain an IBU value for your beer as follows:

1. Using a volumetric pipet, remove 4.0 mL of beer from your jar into a clean 15 mL centrifuge tube. Add 0.5 mL of 3 M HCl to the tube. Cap the tube and invert slowly to mix contents. This step protonates the acids, allowing them to be extracted into an organic solvent.
2. The next step should be done in the fume hood, wearing gloves. Using a volumetric pipet, add 8.0 mL of isooctane to the tube. Cap and shake vigorously for 5 minutes. Carefully vent the cap often to release pressure, but DO NOT SPILL the contents. (Isooctane is volatile and hazardous to inhale.)

3. To ensure proper separation of the organic and aqueous layers, centrifuge the capped tubes at 3000 rpm for 5 min in the DuPont benchtop centrifuge. A protein emulsion should form between the lower water layer and the upper isooctane layer.
4. Carefully remove the clear top layer containing the iso-alpha-acids into a fresh tube. Do not disturb the emulsion when you remove the top layer.
5. Transfer 3 mL of the pure top layer to a quartz cuvette for measurement of absorbance at 275 nm. Blank with pure isooctane. (Share this blank around to minimize chemical waste.) Record the absorbance value of the extracted beer. If the absorbance value is greater than about 1.2, you should dilute your sample by a factor of two with isooctane and read the absorbance again.
6. Multiply the absorbance value by 50 to calculate the IBU.
7. Dispose of any sample containing isooctane in the flammable waste.

2. Beer Color (SRM)

Another objective characteristic of beer is color relative to an established scale- this is the Standard Reference Method (SRM). A beer color chart can be matched by eye, or the determination can be made spectrophotometrically. Obtain an SRM value for your beer as follows:

1. Transfer about 1 mL of the original beer sample to a clean cuvette and measure the absorbance at 430 nm. If your beer is cloudy, centrifuge it first. If your beer is dark, it may require dilution for the reading to be on scale.
2. Calculate color using the SRM scale:

$$SRM = A_{430} \times 12.7 \times D$$

where D is the dilution factor.

3. Use the chart below to double-check your spectrophotometric data. Is the color consistent with the predicted beer type, given what you set out to brew?

Beer Colors and SRM Value		
Color Swatch	SRM	Color
	2	Pale Straw
	3	Straw
	4	Pale Gold
	6	Deep Gold
	9	Pale Amber
	12	Medium Amber
	15	Deep Amber
	18	Amber-Brown
	20	Brown
	24	Ruby Brown
	30	Deep Brown
	40	Black

Beer Style SRM Color Ranges	
Style	SRM Range
Pilsner	2 - 7
Witbier, Berliner Weisse	2 - 4
Belgian Strong Ale	4 - 7
Maibock	4 - 10
Vienna Lager	7 - 14
Oktoberfest	4 - 12
American Pale Ale	6 - 14
Pale Ale	5 - 14
English Golden Ale	4 - 8
Bavarian Weizen	4 - 10
Bitter, ESB	8 - 14
Märzen	7 - 15
Imperial Pale Ale	5 - 11
Bière de Garde	6 - 13
Dunkel Weizen	9 - 13
Amber Ale	11 - 18
English Brown Ale	12 - 22
Bock	15 - 30
Porter	20 - 40
Oatmeal Stout	25 - 40
Baltic Porter	17 - 40
Foreign Stout	30 - 65
Imperial Stout	50 - 80

Source: Tasting Beer by Randy Mosher

3. Alcohol Content (ABV)

Although refractometry or specific gravity can be used to assess ABV with a "before" and "after" measurement, we are using a spectrophotometric determination of ethanol content in this experiment. This

method relies on a color change when orange dichromate ion ($Cr_2O_7^{2-}$) is reduced to green chromic ion (Cr^{3+}). Ethanol acts as the reducing agent in the reaction as follows:

$$2\ Cr_2O_7^{2-} + 3\ CH_3CH_2OH + 16\ H^+ \rightarrow 4\ Cr^{3+} + 3\ CH_3COOH + 11\ H_2O$$

Obtain an ABV value for your beer as follows:

1. Perform an extraction with Tri-n-butyl phosphate (TBP) by mixing 1.0 mL of TBP and 1.0 mL of beer in a microcentrifuge tube and then vortexing vigorously for 1 min.
2. Centrifuge at 3,450 x g for 5 min to separate the phases. Ethanol will be in the upper organic layer, which should be clear and transparent.
3. Transfer 500 µL of the upper phase to a new microcentrifuge and add 500 µL of dichromate reagent (10% w/v of $K_2Cr_2O_7$ in 5 M of H_2SO_4). Vortex vigorously for 1 min.
4. Incubate for 10 min at room temperature to allow the redox reaction to occur.
5. Add 270 µL of deionized water to appropriate wells of a 96-well plate
6. Carefully remove 30 µL of the reaction's lower oxidized phase and add it to the well plate, gently mix with pipet tip.
7. Measure absorbance at 595 nm using the endpoint setting on the plate reader.
8. Using the standard curve of % ethanol versus A_{595} to determine the % ethanol in your beer.

Part J. Bottling the Beer

1. The yeast will mostly be settled at the bottom of the jar. Carefully decant the wort into a clean jar. Try to avoid getting too much of the yeast sediment into the new jar.
2. Add a teaspoon of sucrose to the decanted wort and stir to dissolve. The small amount of yeast remaining will ferment this sugar, producing CO_2 that carbonates the beer. This process is called "conditioning."
3. Use a funnel to dispense the contents into 2 beer bottles. Try to get approximately the same amount in each bottle. Label the bottles with your own "special" beer label!
4. Cap the bottles with the bottle capper and incubate in the dark at room temperature for 3 weeks.

Week 6 (actually 3 weeks later)

Part K. Subjective Evaluation of Beer Quality

Proceed with this part only if your beer seems palatable! If your ethanol content was very low or if the beer smells bad, do not taste! Although you can still assess the aroma, it probably will not fall into any of the categories on the scorecard.

If your beer passes the sniff test, find at least three volunteers of legal age, and have them assess the final product using the Beer scorecard in Appendix 1.

References

S. Banos et al. (2018) A comprehensive fungi-specific 18S rRNA gene sequence primer toolkit suited for diverse research issues and sequencing platforms. *BMC Microbiol 18*, 190. doi.org/10.1186/s12866-018-1331-4

B. Gibson, J.-M. A. Geertman, C. T. Hittinger, K. Krogerus, D. Libkind, E. J. Louis, F. Magalhães, J. P. Sampaio (2017) New yeasts—new brews: modern approaches to brewing yeast design and development. *FEMS Yeast Research 17*. https://doi.org/10.1093/femsyr/fox038

B. C. Hamper, J. W. Meisel (2020) Introducing nonscience majors to science literacy via a laboratory and lecture beer brewing course. *J. Chem. Educ. 97*, 1289– 1294.

T. Meier-Dörnber, M. Hutzler, M. Michel, F.-J. Methner, F. Jacob (2017) The importance of a comparative characterization of *Saccharomyces cerevisiae* and *Saccharomyces pastorianus* strains for brewing. *Fermentation 3*, 41. https://doi.org/10.3390/fermentation3030041

M. Sriariyanun, P. Mutrakulcharoen, S. Tepaamorndech, K. Cheenkachorn, K. A. Rattanaporn (2019) Rapid spectrophotometric method for quantitative determination of ethanol in fermentation products. *Orient J Chem 35* (2).

F. A. Thesseling et al. (2019) A hands-on guide to brewing and analyzing beer in the laboratory. *Current Protocols in Microbiology 54*, e91. Doi: 10.1002/cpmc.91

ASSIGNMENT

Working with your partner, prepare a research poster on your work. Choose an interesting experimental question that can be answered with your data (or the class data), using the literature to set the context. (It's not sufficient to have the question be, "Can we brew beer with wild yeast?")

Appendix 1.

Beer Scorecard

NAME OF BEER__________________ **YOUR NAME__________________**

AROMA

CARAMEL/CHOCOLATE

FLORAL

HERBS/SPICES

FRUITY

SMOKY

VINEGARY

EARTHY/WOODSY

MALTY/YEASTY

MOUTHFEEL

ASTRINGENT (MOUTHPUCKERING, DRY)

FLAT

FIZZY

METALLIC

SMOOTH

WARMING

FLAVOR

SOUR/TART

BITTER

FLORAL

FRUITY

MALTY/YEASTY

SMOKY

CLOVES/SPICES

OVERALL RATING

☆ ☆ ☆ ☆ ☆

COMMENTS:
