# Supplementary material for: Fermentation Gone Wild: A Biochemistry Laboratory Experiment
Source: J Chem Educ. 2023 Jul 26;100(8):3076–80. doi: 10.1021/acs.jchemed.3c00499 (PMC10413941; doi:10.1021/acs.jchemed.3c00499)
Supplement: Supplementary file 5 — ed3c00499_si_005.pdf [file ed3c00499_si_005.pdf]

Backyard Brewery: Brewing Beer with Wild Yeast

Student 1 and Student 2

Department of Chemistry, Colby College, Waterville, ME 04901

Abstract

The history of fermentation involved using natural yeast sources to make alcoholic drinks.[2,4,8,9] This experiment used two experimental yeasts: *Zygotorulaspora florentina* and *Aureobasidium pullulans*. Both yeasts have shown positive results in previous studies. The experiment aimed to produce beer using wild yeast fermentation. This experiment involved selecting yeast colonies from a chosen plant or area and conducting PCR assays to determine the yeast species. A three-week fermentation process was carried out to evaluate the beer characteristics, including IBU, SRM, and ABV. Qualitative analysis was also conducted to assess mouthfeel, flavor, aroma, and rating. However, the ABV levels of both yeasts fell short of the standard 4%. Despite this, both exhibited successful fermentation and produced ethanol. Quality was positive, with "fruity" and "spicy/fruits/flowers" aromas for Z. florentina and A. pullulans. Flavors for *Z. florentina* and *A. pullulans* were "fruity/sweet" and "sweet" respectively. A gassy mouthfeel was reported for both.

Introduction

History of Fermentation

- Historians suggest that humans have been using fermentation for food since 6000 BCE.[2]
- Alcoholic beverages were produced through fermentation using sources such as fruit and rice with naturally occurring yeast [4,8]
- The discovery of the mechanism of yeast's ability to create CO_2 and ethanol from sugar was made in 1860.[9]

Experimental Strain: *Zygotorulaspora florentina*

- The species may be isolated from plants and grapes [5]
- It has been studied as an alternative form of yeast in fermentation [6]
  - The study found that including it positively increased the wine quality, especially when mixed with the typical brewer's yeast species used in fermentation [6]
- It has been shown to utilize both maltose and maltotriose in fermentation [5]

Experimental Strain: *Aureobasidium pullulans*

- Black yeast which can produce a dark pigment.[3]
- Commonly occurring yeast in nature due to its resistance to pH changes caused by different climates worldwide.[3]
- It has been found in various grape juices, leading to a study on its effects on ethanol production.[7]
- A study found that it can be used as a substitute for *S. cerevisiae* yeast in alcoholic beverages.[7]

Process Overview

- Wrangling of yeast – a plant/area of our choosing
- Culture and growth of selected sample
- Sequencing to determine species of yeast
- Potential for phenolic off flavors (ferulic acid assay)
- 3 week fermentation
- Beer Characteristics Evaluated:
  - IBU – an international bitterness scale, ranges from 5 (low bitterness) to 120 (high bitterness)
  - SRM – Standard Reference Method, use of a chart to determine the type of ale and coloring, SRM can be used to assess ale type, scale ranges from 2–7 (Pilsner) to 50–80 (Imperial Stout)
  - ABV – alcohol content
- Bottled and left for 3 more weeks
- Qualitative analysis of final product

Experimental Goal

The overarching goal of this project was to brew our own beers using yeast obtained from the wild.

Identification of Wild Yeast

Sample	Source	GPS Coordinates	Yeast Strain	Culture Aroma	POF (+/-)
Bey's Beer	Crabapple	44.563477, -69.662978	*Zygotorulaspora florentina*	Fruity	(-)
Sarah's Beer	Rosehip	44.56,-69.66	*Aureobasidium pullulans*	Sweet/Smoky Honey	(+)

Description	Scientific Name	Max Score	Total Score	Query Cover	E value	Per. Ident	Acc. Len	Accession
Z.florentinus 18S ribosomal RNA	Zygotorulaspor...	941	941	100%	0.0	100.00%	1768	X91086.1
Zygosaccharomyces sp. IFO 11069 gene for 18S rRNA, partial sequence	Zygosaccharom...	924	924	100%	0.0	99.41%	1754	AB087385.1
Uncultured fungus clone TEU7-57 18S ribosomal RNA gene, partial sequence	uncultured fungus	913	913	100%	0.0	99.02%	1736	KC337084.1
Zygotorulaspora mrakii 18S ribosomal RNA (HG535_RND18a), rRNA	Zygotorulaspor...	913	913	100%	0.0	99.02%	1800	XR_005101481.1
Zygotorulaspora mrakii 18S ribosomal RNA (HG535_RND18e), rRNA	Zygotorulaspor...	913	913	100%	0.0	99.02%	1800	XR_005101485.1
Zygotorulaspora mrakii 18S ribosomal RNA (HG535_RND18d), rRNA	Zygotorulaspor...	913	913	100%	0.0	99.02%	1800	XR_005101484.1
Zygotorulaspora mrakii 18S ribosomal RNA (HG535_RND18c), rRNA	Zygotorulaspor...	913	913	100%	0.0	99.02%	1800	XR_005101483.1

Description	Scientific Name	Max Score	Total Score	Query Cover	E value	Per. Ident	Acc. Len	Accession
Aureobasidium pullulans strain DSM 3497 18S ribosomal RNA gene, partial sequence	Aureobasidium...	665	1311	100%	0.0	100.00%	1708	EU682925.1
Dothioraceae sp. strain LSU0866 small subunit ribosomal RNA gene, partial sequence	Dothioraceae sp.	664	1296	98%	0.0	100.00%	1028	MT000356.1
Aureobasidium sp. isolate 6C small subunit ribosomal RNA gene, partial sequence	Aureobasidium...	664	1315	100%	0.0	100.00%	1653	MN995480.1
Aureobasidium sp. isolate 3CYEAST small subunit ribosomal RNA gene, partial sequence	Aureobasidium...	664	1315	100%	0.0	100.00%	1655	MN995476.1
Aureobasidium sp. strain SM15UFAM small subunit ribosomal RNA gene, partial sequence; interna...	Aureobasidium...	664	1315	100%	0.0	100.00%	2841	MN268776.1
Aureobasidium pullulans strain MFLUCC 17-1256 18S ribosomal RNA gene, partial sequence	Aureobasidium...	664	1257	95%	0.0	100.00%	702	MG812620.1
Aureobasidium pullulans strain MFLUCC 17-1231 18S ribosomal RNA gene, partial sequence	Aureobasidium...	664	1257	95%	0.0	100.00%	702	MG812619.1

- Location of yeast source identified via GPS coordinates
- The 18S rRNA gene was amplified via PCR and subsequently sequenced
- NCBI BLAST was used to identify yeast strain[1]

Zygotorulaspora florentina:
- No potential for phenolic off flavors

Aureobasidium pullulans:
- Potential for phenolic off flavors

Beer Characteristics

- Both of our beers had low alcohol contents
  - This was interesting considering one of our strains is used in brewing
- Both had similar IBU values for bitterness
- Relatively similar SRM values

- Overall average ABV of all beers brewed was similar to the average alcohol content of a beer (around 4%)
- There were large deviations in all statistics due to the small sample size for some genera

Beer	ABV	SRM (Color)	IBU (Bitterness)	Beer Type
Bey's Beer	0.79	20.1	6.5	English Brown Ale
Sarah's Beer	1.63	17.69	6.9	Amber Ale

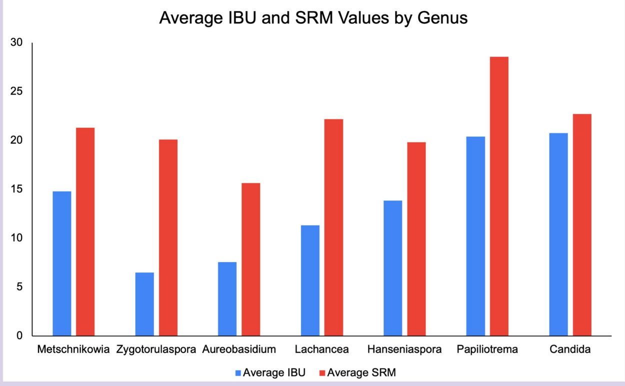

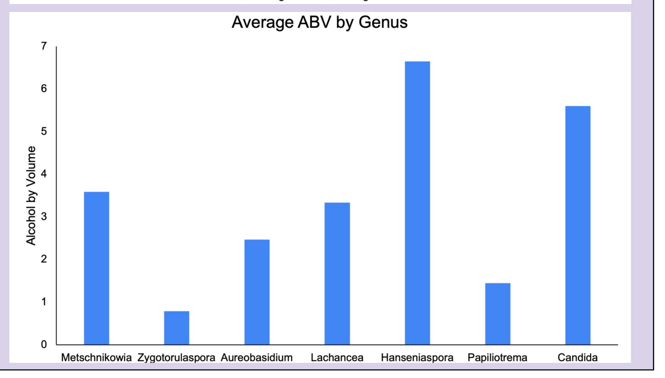

Qualitative Analysis

- Yeast from 8 separate genera were used for fermentation
  - Yeast of some genera were preferred over the Brewer's yeast control
  - Sample size was limited
  - Too small to draw definitive conclusions
- Sample size was also too small to draw conclusions regarding relationship between rating and yeast source
- Despite using different strains and sources, our beers yielded similar reports across the board

Z. florentina average score: 3.91
A. pullans average score: 3.6

Genus	Sample Size
Metschnikowia	3
Saccharomyces	2
Zygotorulaspora	1
Aureobasidium	4
Lachancea	2
Hanseniaspora	2
Papiliotrema	1
Candida	1

Source	Sample Size
Crabapple	3
Control	2
Juniper berry	1
Sour Cherry	2
Buckthorn	2
Bitter sweet	1
Birch	2
Rosehip	2
Blackberry	1
Chestnut Oak	1

Beer	Top Reported Mouthfeel	Top Reported Aroma	Top Reported Flavor
Bey's Beer	Gassy	Fruity	Fruity / Sweet
Sarah's Beer	Gassy	Fruits/Flowers/Spice	Sweet

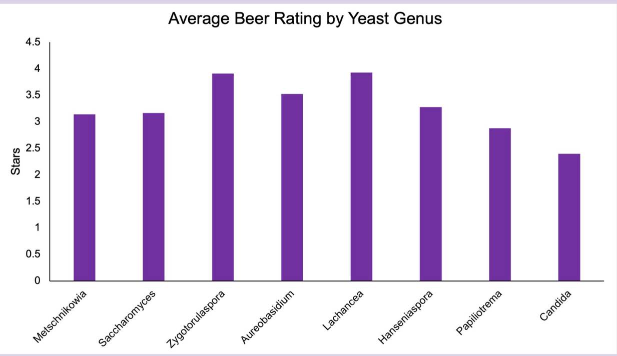

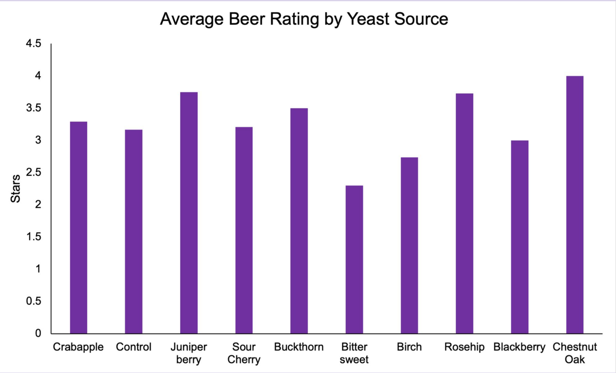

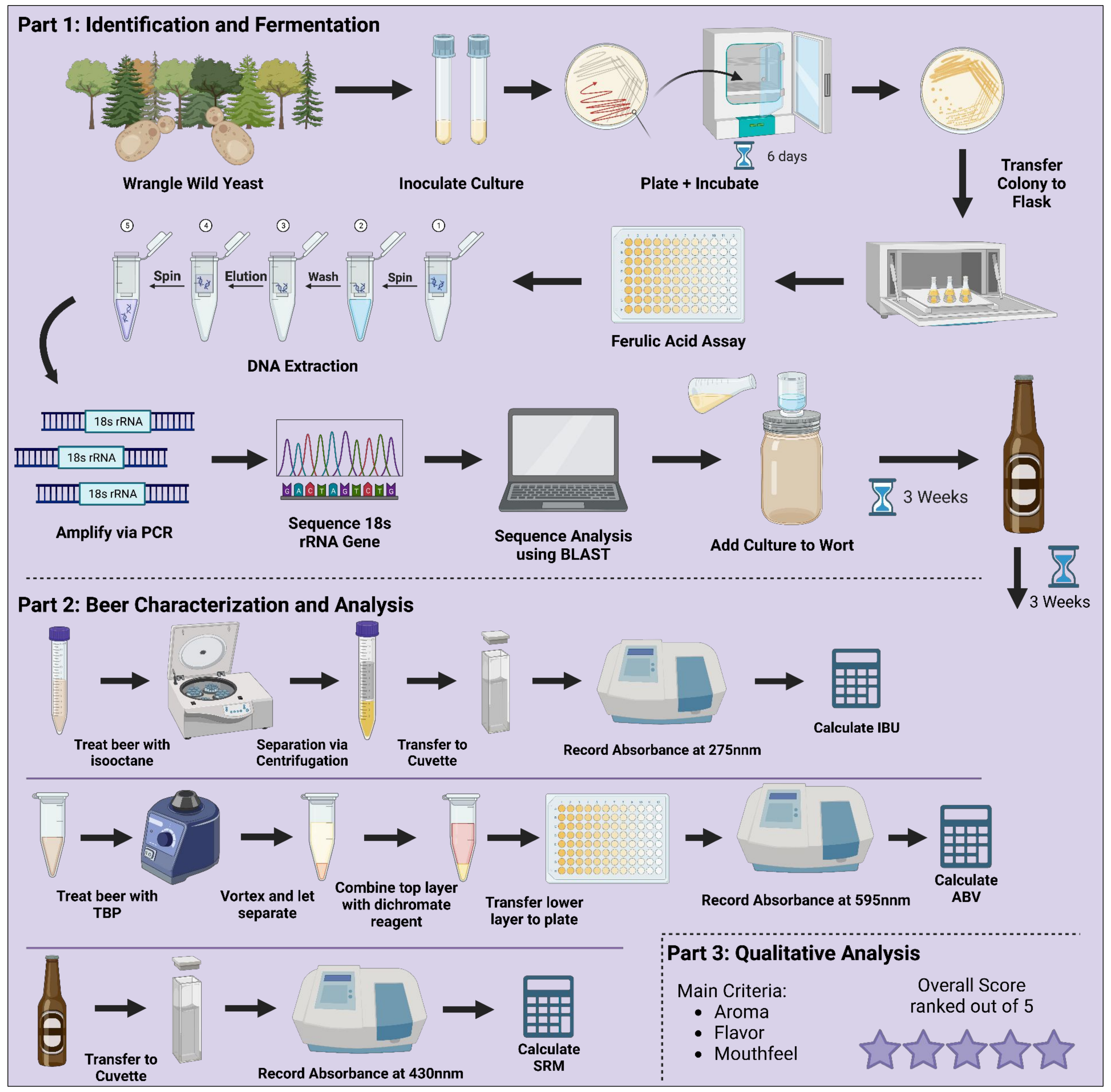

Discussion

- Neither strain was successful in creating a standard beer alcohol content
  - Standard is approximately 4% ABV whereas the *Zygotorulaspora florentina* and *Aureobasidium pullulans* produced an ABV of 0.79% and 1.63% respectively
- Both exhibited successful fermentation despite not reaching standards
  - ABV calculations above 0% demonstrates ethanol was produced throughout the fermentation process
- Overall quality of the beers was positive
  - Both had a top reporter of "gassy" for mouthfeel
  - The top reported aroma for *Zygotorulaspora florentina* and *Aureobasidium pullulans* was "fruity" and "spicy/fruits/flowers" respectively
  - The top reported flavor for *Zygotorulaspora florentina* and *Aureobasidium pullulans* was "fruity/sweet" and "sweet" respectively
- Even though there were many different types of yeast, all provided positive scores for the subjective measurements and could be used in subsequent beer production

References

(1)
Zhang, Z.; Schwartz, S.; Wagner, L.; Miller, W. A Greedy Algorithm for Aligning DNA Sequences. *J Comput Biol* **2000**, *7* (1–2), 203–214. https://doi.org/10.1089/10665270050081478.

(2)
Beyond Sauerkraut: A Brief History of Fermented Foods. https://www.lhf.org/2014/03/beyond-sauerkraut-a-brief-history-of-fermented-foods/ (accessed 2023-04-27).

(3)
Chi, Z.; Wang, F.; Chi, Z.; Yue, L.; Liu, G.; Zhang, T. Bioproducts from Aureobasidium Pullulans, a Biotechnologically Important Yeast. *Appl Microbiol Biotechnol* **2009**, *82* (5), 793–804. https://doi.org/10.1007/s00253-009-1882-2.

(4)
Bread, beer and wine: Yeast domestication in the Saccharomyces sensu stricto complex | Elsevier Enhanced Reader. https://doi.org/10.1016/j.crvi.2010.12.016.

(5)
Nikulin, J.; Eerikäinen, R.; Hutzler, M.; Gibson, B. Brewing Characteristics of the Maltotriose-Positive Yeast Zygotorulaspora Florentina Isolated from Oak. *Beverages* **2020**, *6* (4), 1–19. https://doi.org/10.3390/beverages6040058.

(6)
Lencioni, L.; Romani, C.; Gobbi, M.; Comitini, F.; Ciani, M.; Domizio, P. Controlled Mixed Fermentation at Winery Scale Using Zygotorulaspora Florentina and Saccharomyces Cerevisiae. *International Journal of Food Microbiology* **2016**, *234*, 36–44. https://doi.org/10.1016/j.ijfoodmicro.2016.06.004.

(7)
Onetto, C. A.; Borneman, A. R.; Schmidt, S. A. Investigating the Effects of Aureobasidium Pullulans on Grape Juice Composition and Fermentation. *Food Microbiology* **2020**, *90*, 103451. https://doi.org/10.1016/j.fm.2020.103451.

(8)
Foroutan, R. *The History and Health Benefits of Fermented Food - Food & Nutrition Magazine.* https://foodandnutrition.org/winter-2012/history-health-benefits-fermented-food/ (accessed 2023-04-27).

(9)
Yeast, Fermentation, Beer, Wine | Learn Science at Scitable. https://www.nature.com/scitable/topicpage/yeast-fermentation-and-the-making-of-beer-14372813/ (accessed 2023-04-27).

Acknowledgements

We would like to thank Professor Julie Millard, our Biochemistry laboratory and lecture instructor, for her aid throughout the lab process. She ordered the primers and sent them to the company that conducted the Sanger sequencing. Additionally, we would also like to thank Victoria Hepburn for helping prepare some of the reagents used throughout the procedure. Furthermore, we would like to thank Colby College and the donors who allowed these lab materials to be purchased.
