# Supplementary material for: Fermentation Gone Wild: A Biochemistry Laboratory Experiment
Source: J Chem Educ. 2023 Jul 26;100(8):3076–80. doi: 10.1021/acs.jchemed.3c00499 (PMC10413941; doi:10.1021/acs.jchemed.3c00499)
Supplement: Supplementary file 6 — ed3c00499_si_006.pdf [file ed3c00499_si_006.pdf]

Brewing the Perfect Beer: Exploring the Effects of Yeast Strains on Flavor, Aroma, and Alcohol Content

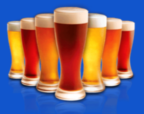

Student A, Student B

Department of Chemistry, Colby College, Waterville, ME 04901

Introduction

- Fermentation with yeast has been used to make bread and alcohol beverages for thousands of years.[1]
- Different yeast strains can produce beer with high variations of flavor and aroma resulting in unique beer types. Through glycolysis, yeast convert glucose from sugars into cytosolic pyruvate. Under anaerobic conditions, pyruvate is oxidized into acetaldehyde, and acetaldehyde is reduced into ethanol.
- Both *Metschnikowia pulcherrima* and *Aureobasidium pullulans* are common yeast strains found on different fruits and surfaces.[2] Fermentation using *Metschnikowia pulcherrima* has shown to have low levels of acidity and comparable alcohol contents to beer (<10%).[3] However, the fermentation using *Aureobasidium pullulans* has not been extensively studied.
- The 18S ribosomal gene was used as a genetic marker for yeast identification.
- In this experiment, we investigated the differences between two different yeast strains, *Metschnikowia pulcherrima* and *Aureobasidium pullulans*, on the sensory characteristics and alcohol content of beer.
- Understanding the impact of yeast strain on beer characteristics can inform aspiring brewers. Additionally, this study can be used to educate students on the biochemistry of brewing and yeast fermentation

Methods

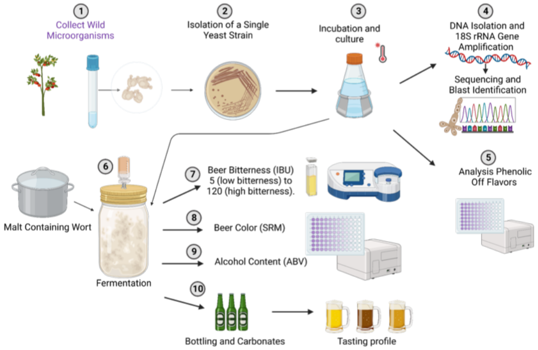

Figure 1. Experimental flow chart of yeast identification and culturing, and beer fermentation and characterization.

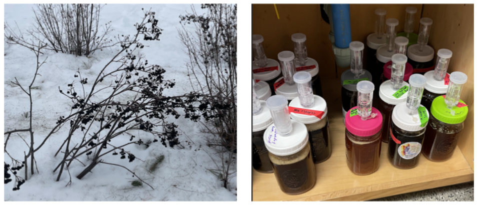

Figure 2. Yeast samples collected from *Rhamnus cathartica* (left), *Rosa canina L.*, *Ilex verticillata*, and *Prunus cerasus*. Yeast strains, *Metschnikowia pulcherrima* and *Aureobasidium pullulans*, were used for fermentation. The experimental setup using Mason jars and fermentation lids are shown (right). Jars were stored at 25°C without light.

Results. Yeast Characterization

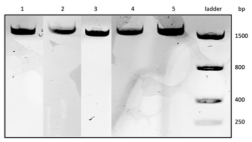

Figure 3. 1.2%FlashGel of the 18S ribosomal RNA gene PCR products of Samples 1-5 with the FlashGel QuantLadder. The 18S ribosomal RNA gene is 1613 bp.

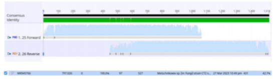

Figure 4. Alignment of 18S rRNA sequence from the yeast isolate of Sample 4 with known yeast sequences.

- DNA was isolated and amplified from the yeast strains obtained from different fruit sources using PCR.
- The 18S ribosomal RNA gene is 1613 bp in length. All samples suggested the PCR product contained the 18S ribosomal RNA gene (Figure 3).
- The sequence alignments of the yeast isolates with known yeast sequences for samples 1-5 all had %identity of over 99% (Figure 4). Sequence alignments for Samples 1-3 and 5 were not shown.
- The alignment revealed 100% sequence identity with *Metschnikowia sp.*, indicating that the yeast isolate belongs to this species.

Research Question

Which strain of yeast, *Metschnikowia pulcherrima* or *Aureobasidium pullulans*, used for fermentation will produce a higher quality beer?

Results. Beer Characterization

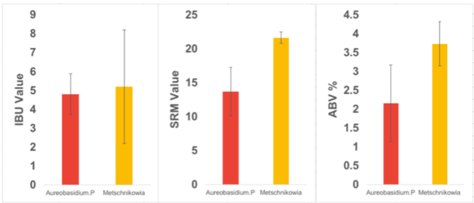

Figure 5. IBU, SRM, and ABV characterization tests for beer fermented with *Aureobasidium pullulans (red)* and *Metschnikowia pulcherrima (yellow)*. Standard deviation was used for error (n=3 for *Aureobasidium p.*) (n=2 for *Metschnikowia p.*)

- IBU measures the bitterness of beer by determining the concentration of iso-alpha acids. Higher IBU values indicate a more bitter taste.
- SRM is a color scale in determining its flavor profile. Higher SRM values indicate a darker color.
- Alcohol by volume (ABV)% measures alcohol content. The higher the ABV%, the more ethanol production from yeast in the beer.
- *Metschnikowia pulcherrima* had higher IBU, SRM, and ABV% values compared to *Aureobasidium pullulans* (Figure 5).
- A ferulic acid test for Phenolic off flavors is performed to assess the ability of our yeast strain to convert ferulic acid to 4-vinyl guaiacol (4-VG), a phenolic compound that contributes to off-flavors in some beer styles and is desired in others. Unfortunately, the test data was lost and no results were obtained for this experiment.

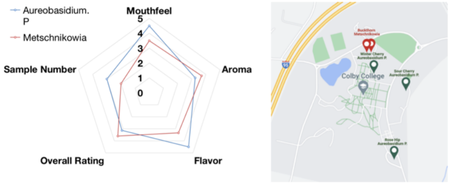

Figure 6. Evaluation rating chart and fruit location. Mouthfeel, aroma, flavor, overall rating, and sample size were shown on the left. The scales were arbitrary and based on online consumer preference.[4,5] The locations of where the fruits and yeast strains were collected were shown on the right. (n=3 for *Aureobasidium p.*) (n=2 for *Metschnikowia p.*)

- Figure 4 presented an evaluation rating chart based on mouthfeel, aroma, flavor, and overall rating, and sample size.
- The scales used in the chart are arbitrary and based on online consumer preferences.[4,5]
- The map indicated the locations of where the fruit and yeast strains were collected (Figure 6).
- *Aureobasidium p.* had a slightly greater mouthfeel and flavor preference compared to *Metschnikowia p.*
- However, *Metschnikowia* had a greater aroma and overall rating than *Aureobasidium p.*
- Both strains had a small sample size indicating the need for further experimentation

Conclusions

- Bitterness, color, and alcohol content for beer fermented with *Metschnikowia pulcherrima* were not significantly different to beer fermented with *Aureobasidium pullulans*
- Limitations to the experiment:
  - Sample size was too small
  - A ferulic acid test was not conducted to compare beer fermented with different yeast
- However, beer fermented with *Metschnikowia pulcherrima* was ranked higher in mouthfeel and flavor, whereas beer fermented with *Aureobasidium pullulans* was ranked higher in aroma and overall rating

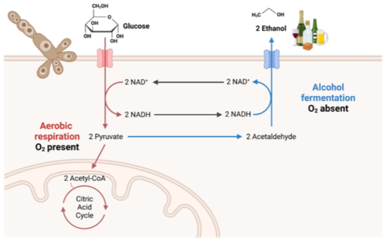

Figure 7. Glucose metabolism under aerobic and anaerobic conditions. Under anaerobic conditions, yeast convert cytosolic pyruvate into ethanol to produce alcoholic beverages. Under aerobic conditions, pyruvate is transported into the mitochondrial matrix and reduced into acetyl-CoA for the citric acid cycle.

References

1. Kitagaki, H., & Kitamoto, K. (2013). Breeding Research on Sake Yeasts in Japan: History, Recent Technological Advances, and Future Perspectives. *Annual Review of Food Science and Technology*, *4*(1), 215–235. https://doi.org/10.1146/annurev-food-030212-182545

2. Chowdhary, A.; Perfect, J.; De Hoog, G. S. Black Molds and Melanized Yeasts Pathogenic to Humans. *Cold Spring Harb. Perspect. Med.* **2015**, *5* (8), a019570. https://doi.org/10.1101/cshperspect.a019570.

3. Mestre Furlani, M. V.; Maturano, Y. P.; Combina, M.; Mercado, L. A.; Toro, M. E.; Vazquez, F. Selection of Non-Saccharomyces Yeasts to Be Used in Grape Musts with High Alcoholic Potential: A Strategy to Obtain Wines with Reduced Ethanol Content. *FEMS Yeast Res.* **2017**, *17* (2). https://doi.org/10.1093/femsyr/fox010.

4. Lerro, M., Marotta, G., & Nazzaro, C. (2020). Measuring consumers' preferences for craft beer attributes through Best-Worst Scaling. Agricultural and Food Economics, 8(1). https://doi.org/10.1186/s40100-019-0138-4

5. "The 7 Flavor Categories of Beer: What They Are, How to Pair Them." The Splendid Table, 21 Mar. 2013, https://www.splendidtable.org/story/2013/03/21/the-7-flavor-categories-of-beer-what-they-are-how-to-pair-them.
